# Age at menarche and prevalence of preterm birth: Results from the Healthy Baby Cohort study

**DOI:** 10.1038/s41598-017-12817-2

**Published:** 2017-10-03

**Authors:** Hui Li, Lulu Song, Lijun Shen, Bingqing Liu, Xiaoxuan Zheng, Lina Zhang, Yuanyuan Li, Wei Xia, Bin Lu, Bin Zhang, Aifen Zhou, Zhongqiang Cao, Youjie Wang, Shunqing Xu

**Affiliations:** 1 Department of Maternal and Child Health, School of Public Health, Tongji Medical College, Huazhong University of Science and Technology, Wuhan, 430030 Hubei China; 2 Key Laboratory of Environment and Health, Ministry of Education & Ministry of Environmental Protection, and State Key Laboratory of Environmental Health, School of Public Health, Tongji Medical College, Huazhong University of Science and Technology, Wuhan, 430030 Hubei China; 3 Woman and Children Medical and Healthcare Center of Wuhan, Wuhan, 430030 Hubei China

## Abstract

Little is known about the impact of age at menarche on preterm birth. The aim of this study was to examine the association between age at menarche and preterm birth. A total of 11,016 Chinese women who gave birth to live singleton infants were recruited from the Healthy Baby Cohort between 2012 and 2014 in the province of Hubei, China. Age at menarche was reported via face-to-face interviews and was categorized into five groups (≤11, 12, 13, 14 and ≥15 years). Gestational age was estimated using maternal last menstrual period. Preterm birth was defined as delivering a live singleton infant at <37 weeks’ gestational age. Logistic regression was used to estimate odds ratios (ORs) and 95% confidence intervals (CIs). Earlier menarche (≤11 years) was associated with an increased prevalence of preterm birth (OR: 1.67, 95% CI: 1.18, 2.36) compared with menarche age at 13 years after controlling for the potential confounders. The findings of our study suggested that a history of earlier menarche might be useful for identifying women at higher risk of preterm birth.

## Introduction

Preterm birth (PTB, <37 completed weeks of gestation), an important adverse pregnancy outcome, affects approximately 14.9 million infants worldwide in 2010^1^. Preterm infants are at increased risk of developing neonatal and long-term complications^2^. Thus, identifying women at risk of PTB at an early life stage may allow early health monitoring and intervention.

Menarche, the age at onset of first menstruation, is an indicator of puberty^3^. Secular trends in earlier menarche have been observed worldwide^4–6^. Potential health effects of age at menarche have received a great deal of attention. Earlier menarche has been linked to several adverse health consequences in later life, such as breast cancer^7^, type 2 diabetes^8^, and cardiovascular diseases^9^. In addition, earlier menarche has been associated with adverse pregnancy outcomes, such as ectopic pregnancies^10^, miscarriage^11^, and low birth weight^12^. However, little is known about the influence of age at menarche on PTB. Previous studies have suggested that earlier menarche was associated with higher estradiol levels^13,14^, elevated C-reactive protein levels^15^, and increased plasma glucose levels in the adulthood^16,17^. These changes were reported to be related to an increased risk of PTB^18–23^. In addition, earlier menarche was associated with PTB risk factors, such as obesity^24,25^, infection^26,27^, and psychological stress^28,29^. To our knowledge, except for one study reported no significant association between age at menarche and PTB among 2115 women from the 1958 British birth cohort study^30^, we are not aware of any epidemiological studies that have investigated the relationship between them.

The aim of this study was to examine the association between age at menarche and prevalence of PTB among Chinese women, using data from the Healthy Baby Cohort (HBC) study. We hypothesized that earlier menarche was associated with an increased prevalence of PTB.

## Results

### Characteristics of the participants

The present study included 11,016 live singleton infants, 653 (5.9%) were born preterm. The mean gestational age was 39.1 ± 1.4 weeks. The mean maternal age was 28.2 ± 3.7 years and the mean age at menarche was 13.2 ± 1.2 years. The proportion of women who reported their menarche at ages ≤11, 12, 13, 14 and ≥15 years was 5.1%, 23.2%, 35.7%, 23.8% and 12.2%, respectively. Characteristics of the participants are showed in Table 1. Women with earlier menarche were more likely to have higher pre-pregnancy body mass index (BMI), shorter gestational age, and higher prevalence of diabetes and hypertension during pregnancy (all *P* for trend <0.05).

**Table 1:** Characteristics of the study population according to age at menarche.

Characteristics	Age at menarche (years)					*P* for trend^a^
	≤11 (mean = 10.8)	12	13	14	≥15 (mean = 15.5)	
No. of participants	559	2557	3937	2617	1346	
Maternal age (years)	28.9 ± 3.5	28.2 ± 3.6	28.1 ± 3.7	28.2 ± 3.6	28.4 ± 4.1	0.219
Pre-pregnancy BMI (kg/m^2^)	21.8 ± 3.1	21.1 ± 2.9	20.4 ± 2.6	20.3 ± 2.5	20.0 ± 2.4	<0.001
Gestational age (days)	272.4 ± 10.8	273.1 ± 9.8	273.7 ± 9.8	274.1 ± 9.6	273.2 ± 10.6	0.004
Educational level						<0.001
High school or below	104 (18.6)	723 (28.3)	1334 (33.9)	877 (33.5)	617 (45.8)	
College or above	455 (81.4)	1831 (71.6)	2601 (66.0)	1740 (66.5)	729 (54.2)	
Missing	0 (0.0)	3 (0.1)	2 (0.1)	0 (0.0)	0 (0.0)	
Occupational status						<0.001
Employed	475 (85.0)	2115 (82.7)	3183 (80.8)	2119 (81.0)	1020 (75.8)	
Unemployed	84 (15.0)	442 (17.3)	764 (19.2)	498 (19.0)	326 (24.2)	
Alcohol consumption before pregnancy						0.886
Yes	14 (2.5)	62 (2.4)	91 (2.3)	55 (2.1)	38 (2.8)	
No	545 (97.5)	2495 (97.6)	3846 (97.7)	2562 (97.9)	1308 (97.2)	
Smoking before pregnancy						0.021
Yes	10 (1.8)	22 (0.9)	31 (0.8)	12 (0.5)	10(0.7)	
No	549 (98.2)	2535 (99.1)	3906 (99.2)	2605 (99.5)	1336 (99.3)	
Passive smoking during pregnancy						0.087
Yes	116 (20.8)	566 (22.1)	850 (21.6)	615 (23.5)	314 (23.3)	
No	443 (79.2)	2265 (88.1)	3087 (78.4)	2002 (76.5)	1032 (76.7)	
Physical activity during pregnancy						0.693
Never or rarely	59 (10.6)	283 (11.1)	439 (11.1)	282 (10.8)	173 (12.9)	
1–2 days/week	61 (10.9)	246 (9.6)	363 (9.2)	231 (8.8)	134 (10.0)	
3–4 days/week	39 (7.0)	202 (7.9)	262 (6.7)	179 (6.8)	102 (7.6)	
5–6 days/week	11 (2.0)	43 (1.7)	52 (1.3)	34 (1.3)	22 (1.6)	
Daily	378 (67.5)	1765 (69.0)	2799 (71.1)	1863 (71.2)	905 (67.2)	
Missing	11 (2.0)	18 (0.7)	22 (0.6)	28 (1.1)	10 (0.7)	
Parity						<0.001
1	497 (88.9)	2258 (88.3)	3371 (85.6)	2202 (84.1)	1068 (79.3)	
≥2	62 (11.1)	299 (11.7)	566 (14.4)	415 (15.9)	278 (20.7)	
The use of assisted reproductive technologies						0.346
Yes	2 (0.4)	23 (0.9)	22 (0.6)	17 (0.6)	6 (0.6)	
No	557 (99.6)	2534 (99.1)	3915 (99.4)	2600 (99.4)	1340 (99.6)	
History of spontaneous abortion						0.873
Yes	25 (4.5)	100 (3.9)	152 (3.9)	104 (4.0)	50 (3.7)	
No	499 (89.2)	2241 (87.7)	3443 (87.4)	2271 (86.8)	1124 (83.5)	
Missing	35 (6.3)	216 (8.4)	342 (8.7)	242 (9.2)	172 (12.8)	
History of induced abortion						0.594
Yes	112 (20.0)	453 (17.7)	750 (19.0)	482 (18.4)	245 (18.2)	
No	412 (73.7)	1888 (73.9)	2845 (72.3)	1893 (72.4)	929 (69.0)	
Missing	35 (6.3)	216 (8.4)	342 (8.7)	242 (9.2)	172 (12.8)	
Diabetes during pregnancy						0.002
Yes	73 (13.1)	235 (9.2)	292 (7.4)	190 (7.3)	115 (8.5)	
No	486 (86.9)	2322 (90.8)	3645 (92.6)	2427 (92.7)	1231 (91.5)	
Hypertension during pregnancy						0.010
Yes	23 (4.1)	93 (3.6)	133 (3.4)	83 (3.2)	29 (2.2)	
No	536 (95.9)	2464 (96.4)	3804 (96.6)	2534 (96.8)	1317 (97.8)	

Data are mean ± SD or number (percentage). Abbreviation: BMI, body mass index. ^a^ *P* values for trend were performed by assigning the median values of each group of age at menarche and fitted this as a continuous variable in separate regression models.

### Association between age at menarche and PTB

Table 2 shows the association between age at menarche and prevalence of PTB. Compared with menarche at age 13 years, earlier menarche (≤11 years) was associated with an increased prevalence of PTB (OR: 1.67, 95% CI: 1.18, 2.36) after adjustment for age, educational level, occupational status, pre-pregnancy BMI, lifestyle factors, the use of assisted reproductive technologies, maternal reproductive history, and maternal history of diseases.

**Table 2:** Adjusted ORs (95% CIs) for the association between age at menarche and PTB.

Age at menarche (years)	PTB events/participants	OR^a^ (95% CI)	OR^b^ (95% CI)
≤11	44/559	1.66 (1.39, 1.97)	1.67 (1.18, 2.36)
12	160/2557	1.16 (1.04, 1.29)	1.19 (0.96, 1.47)
13	226/3937	1.00 (Reference)	1.00 (Reference)
14	130/2617	0.86 (0.77, 0.96)	0.85 (0.68, 1.07)
≥15	93/1346	1.08 (0.95, 1.23)	1.06 (0.82, 1.37)

Abbreviation: OR, odds ratio; CI, confidence intervals; PTB, preterm birth. ^a^Adjusted for maternal age, educational level, and occupational status. ^b^Adjusted for maternal age, educational level, occupational status, pre-pregnancy BMI, alcohol consumption before pregnancy, smoking before pregnancy, passive smoking during pregnancy, physical activity during pregnancy, parity, history of spontaneous abortion, history of induced abortion, the use of assisted reproductive technologies, diabetes during pregnancy, and hypertension during pregnancy.

### Subgroup analyses

The results of subgroup analyses stratified by maternal age, pre-pregnancy BMI, and parity are presented in Table 3, Table 4 and Table 5, respectively. No significant interactions were found between age at menarche and maternal age, pre-pregnancy BMI, and parity on prevalence of PTB (all *P* for interaction >0.05).

**Table 3:** Adjusted ORs (95% CIs) for the association between age at menarche and PTB, stratified by maternal age.

Age at menarche (years)	Maternal age <28 years (n = 5015)		Maternal age ≥28 years (n = 6001)		*P* for interaction
	OR^a^ (95% CI)	OR^b^ (95% CI)	OR^a^ (95% CI)	OR^b^ (95% CI)	
≤11	1.54 (1.15, 2.06)	1.54 (0.86, 2.76)	1.74 (1.39, 2.16)	1.75 (1.13, 2.72)	0.391
12	1.13 (0.96, 1.31)	1.16 (0.85, 1.57)	1.21 (1.05, 1.41)	1.22 (0.91, 1.64)	
13	1.00 (Reference)	1.00 (Reference)	1.00 (Reference)	1.00 (Reference)	
14	0.74 (0.63, 0.88)	0.72 (0.52, 1.01)	0.97 (0.84, 1.14)	0.99 (0.73, 1.35)	
≥15	1.30 (1.09, 1.55)	1.26 (0.88, 1.79)	0.91 (0.75, 1.10)	0.89 (0.61, 1.30)	

Abbreviation: OR, odds ratio; CI, confidence intervals; PTB, preterm birth. ^a^Adjusted for maternal age, educational level, and occupational status. ^b^Adjusted for maternal age, educational level, occupational status, pre-pregnancy BMI, alcohol consumption before pregnancy, smoking before pregnancy, passive smoking during pregnancy, physical activity during pregnancy, parity, history of spontaneous abortion, history of induced abortion, the use of assisted reproductive technologies, diabetes during pregnancy, and hypertension during pregnancy.

**Table 4:** Adjusted ORs (95% CIs) for the association between age at menarche and PTB, stratified by pre-pregnancy BMI.

Age at menarche (years)	Pre-pregnancy BMI <20 kg/m^2^ (n = 5305)		Pre-pregnancy BMI ≥20 kg/m^2^ (n = 5711)		*P* for interaction
	OR^a^ (95% CI)	OR^b^ (95% CI)	OR^a^ (95% CI)	OR^b^ (95% CI)	
≤11	1.69 (0.92, 3.10)	1.72 (0.93, 3.19)	1.63 (1.32, 2.02)	1.61 (1.05, 2.46)	0.585
12	1.00 (0.84, 1.80)	1.02 (0.73, 1.42)	1.27 (1.10, 1.46)	1.28 (0.97, 1.70)	
13	1.00 (Reference)	1.00 (Reference)	1.00 (Reference)	1.00 (Reference)	
14	0.90 (0.66, 1.23)	0.93 (0.68, 1.27)	0.81 (0.69, 0.96)	0.80 (0.58, 1.11)	
≥15	1.10 (0.92, 1.32)	1.12 (0.79, 1.57)	1.04 (0.85, 1.26)	1.00 (0.68, 1.48)	

Abbreviation: OR, odds ratio; CI, confidence intervals; PTB, preterm birth; BMI, body mass index. ^a^Adjusted for maternal age, educational level, and occupational status. ^b^Adjusted for maternal age, educational level, occupational status, pre-pregnancy BMI, alcohol consumption before pregnancy, smoking before pregnancy, passive smoking during pregnancy, physical activity during pregnancy, parity, history of spontaneous abortion, history of induced abortion, the use of assisted reproductive technologies, diabetes during pregnancy, and hypertension during pregnancy.

**Table 5:** Adjusted ORs (95% CIs) for the association between age at menarche and PTB, stratified by parity.

Age at menarche (years)	Parity = 1 (n = 9396)		Parity ≥2 (n = 1620)		*P* for interaction
	OR^a^ (95% CI)	OR^b^ (95% CI)	OR^a^ (95% CI)	OR^b^ (95% CI)	
≤11	1.69 (1.39, 2.06)	1.69 (1.15, 2.48)	1.60 (1.06, 2.41)	1.54 (0.67, 3.56)	0.934
12	1.18 (1.05, 1.33)	1.18 (0.93, 1.50)	1.15 (0.74, 1.79)	1.16 (0.72, 1.86)	
13	1.00 (Reference)	1.00 (Reference)	1.00 (Reference)	1.00 (Reference)	
14	0.85 (0.74, 0.97)	0.84 (0.65, 1.09)	0.86 (0.69, 1.08)	0.89 (0.57, 1.39)	
≥15	1.08 (0.93, 1.26)	1.08 (0.80, 1.46)	1.05 (0.82 1.33)	1.00 (0.62, 1.62)	

Abbreviation: OR, odds ratio; CI, confidence intervals; PTB, preterm birth. ^a^Adjusted for maternal age, educational level, and occupational status. ^b^Adjusted for maternal age, educational level, occupational status, alcohol consumption before pregnancy, smoking before pregnancy, passive smoking during pregnancy, physical activity during pregnancy, parity, history of spontaneous abortion, history of induced abortion, the use of assisted reproductive technologies, diabetes during pregnancy, and hypertension during pregnancy.

## Discussion

In the present study, we investigated the association between age at menarche and PTB among Chinese women. We found that earlier menarche was significantly associated with an increased prevalence of PTB after adjustment for potential confounders.

A study of 2115 women from the 1958 British birth cohort study reported no significant relationship between age at menarche and PTB^30^, a finding that was inconsistent with our results. They used retrospectively collected self-reported information on PTB, which may lead to potential recall bias. In addition, the differences in race, sample size, and categories of age at menarche might be potential explanations for the inconsistent findings. More studies are needed to verify the effect of age at menarche on PTB.

Although the potential mechanisms underlying the association between earlier menarche and prevalence of PTB are unclear, our findings are biologically plausible. Women who experienced earlier menarche have higher levels of estradiol in adulthood^13,14^. It has been reported that higher levels of estradiol increased the risk of PTB^18,19^. In addition, earlier menarche was associated with elevated C-reactive protein levels, which is a marker of inflammation^15^. Studies have shown that maternal C-reactive protein levels during pregnancy were positively associated with risk of PTB^20,21^. Furthermore, earlier menarche was associated with metabolic changes including insulin resistance and increased plasma glucose levels^16,17,31^. Previous studies have demonstrated increasing risk of PTB with increasing maternal plasma glucose levels among women without diabetes mellitus^22,23^. We did not found lower prevalence of PTB among women with later menarche (≥15 years). The possible reason is that later menarche may reflect unhealthy medical conditions such as polycystic ovary syndrome (PCOS)^32^. Women with PCOS also have these metabolic problems, which may increase the risk of PTB^33^.

Another possible explanation for the relationship between earlier menarche and PTB might be obesity. Earlier menarche increased the risk of obesity in adulthood^24,25^, which is an important risk factor for PTB^34,35^. In our study, adjustment for pre-pregnancy BMI (as a continuous variable) in the regression models did not alter the significant association between earlier menarche and prevalence of PTB. In addition, we performed a subgroup analysis according to pre-pregnancy BMI; the association between earlier menarche and prevalence of PTB was consistent across subgroup stratified by pre-pregnancy BMI. Therefore, our results suggested that the association between earlier menarche and prevalence of PTB is independent of pre-pregnancy BMI.

This study has many strengths, including the large sample size, and the inclusion of a wide range of potential confounding variables. Moreover, the use of standard questionnaires and medical records enhanced the reliability of the data.

However, there are several limitations of the present study that should be acknowledged. First, age at menarche was assessed retrospectively, which may result in recall bias. However, previous studies indicated that recalled age at menarche reported during adulthood is highly correlated with original childhood data^36,37^. Second, Because of the lack of detailed information on types of PTB (i.e., spontaneous or iatrogenic PTB), we could not estimate the association between age at menarche and specific types of PTB. Third, the gestational age was calculated from maternal last menstrual period (LMP) in our study, which might result in an inaccurate calculation of gestational age and misclassification of PTB events. However, it has been reported that gestational age estimates by LMP and ultrasound were well correlated, and the estimates of gestational age by LMP was generally reliable and valid^38,39^. Fourth, our study was conducted in Chinese population. Due to the ethnic/racial differences in menarche, our findings may not be generalizable to other populations. Fifth, although a variety of potential confounding variables were adjusted, we cannot rule out the residual confounding by other unmeasured factors such as stress, maternal nutritional status and history of PTB. However, given that 85.3% of the participants were primiparous, no adjustment for history of PTB should not pose a problem for this study.

In conclusion, our study suggested that earlier menarche is a risk factor for PTB. A history of earlier menarche may be useful for identifying women at an elevated prevalence of developing PTB. Future studies are needed to confirm this finding and to clarify the underlying mechanisms.

## Methods

### Study participants

The HBC study is an ongoing prospective birth cohort, which was conducted to investigate the environmental and genetic factors that affect child health and development. Between September 2012 and October 2014, a total of 11,311 women who gave birth to live singleton infants were recruited from the Women and Children Medical and Healthcare Center of Wuhan, Hubei province, China. Each participant was required to provide blood and urine samples, and complete a standard questionnaire by face-to-face interviews. In the present study, we excluded women if they gave birth to an infant with a birth defect or if they had missing information on age at menarche. In total, 295 women were excluded, and 11,016 participants were included for the final analysis.

This study was approved by the Medical Ethics committee of the School of Public Health, Tongji Medical College, Huazhong University of Science and Technology. All participants provided written informed consent. All the methods in the present study were carried out in accordance with the approved guidelines.

### Assessment of age at menarche

Information on age at menarche was obtained from questionnaires based on a question: ‘How old were you when you had the first menstrual period?’ In our study, age at menarche was recorded in years. For the present analysis, we categorized age at menarche into five groups (≤11, 12, 13, 14 and ≥15 years).

### Ascertainment of PTB

Gestational age was estimated as the difference between the first day of the LMP and the delivery date. The first day of the LMP was obtained from the prenatal care record during the first antennal care visit (before 12 weeks), and the delivery day were obtained from medical records. PTB was defined as delivering a live singleton infant at <37 weeks’ gestational age.

### Assessment of covariates

Participants was interviewed during the period of institutional delivery by trained nurses in the hospital. Information on socioeconomic characteristics (maternal age at delivery, educational level, and occupational status), and lifestyle factors (alcohol consumption before pregnancy, smoking before pregnancy, passive smoking during pregnancy, and physical activity frequency during pregnancy) was collected by questionnaires. Passive smoking was defined as exposure second-hand smoking during pregnancy (the father or other persons smoking in the household or workplace)^40^. Data on maternal reproductive history (history of spontaneous abortion, history of induced abortion, and parity), maternal history of diseases, and the use of assisted reproductive technologies were obtained from medical records. Parity was defined as self-reporting of the number of live births. Pre-pregnancy weight was self-reported at the first prenatal care visit (usually at the first trimester), and height was measured using a stadiometer at the first prenatal care visit. Pre-pregnancy BMI was calculated as pre-pregnancy weight in kilograms divided by height in meters squared.

### Statistical analysis

Continuous variables were presented as mean ± standard deviation (SD), and categorical variables were expressed as number and percentage. To test for linear trend across categories of age at menarche, we assigned the median values of each group of age at menarche and fitted this as a continuous variable in a separate regression model.

Logistic regression models were used to estimate odds ratios (ORs) and 95% confidence intervals (CIs) for the association between age at menarche and prevalence of PTB. We used menarche age of 13 years as the reference group, because 13 years old is the median age of menarche of the study participants. Covariates for the adjusted models were selected based on their established or potential association with PTB^35,41–43^, including maternal age (continuous), educational level (high school or below, college or above), occupational status (employed or unemployed), pre-pregnancy BMI (continuous), alcohol consumption before pregnancy (yes or no), smoking before pregnancy (yes or no), passive smoking during pregnancy (yes or no), physical activity frequency during pregnancy (never/rarely, 1–2 days/week, 3–4 days/week, 5–6 days/week or daily), history of spontaneous abortion (yes or no), history of induced abortion (yes or no), parity (1 or ≥2), the use of assisted reproductive technologies (yes or no), hypertension during pregnancy (yes or no), and diabetes during pregnancy (yes or no). We performed a multiple imputation analysis to handle missing data^44^. Twenty imputations were used, and all the adjustment variables listed in the regression analysis above were included in the imputation model, along with the outcome (PTB).

To assess a potential effect modification, analyses were stratified by maternal age (<28 or ≥28 years, the median value of age at delivery), and pre-pregnancy BMI (<20 or ≥20 kg/m^2^, the median value of pre-pregnancy BMI) and parity (1 or ≥2). Tests for interaction across subgroup were performed using Wald test.

All statistical analyses were performed with SAS 9.4 software (SAS Institute Inc., Cary, NC, USA). Significance tests were two-tailed and *P* value < 0.05 were considered significant.
